# CUSCO: An Unobtrusive Custom Secure Audio-Visual Recording System for Ambient Assisted Living

**DOI:** 10.3390/s24051506

**Published:** 2024-02-26

**Authors:** Pierre Albert, Fasih Haider, Saturnino Luz

**Affiliations:** 1 National Institute for Public Health and the Environment, 3721 MA Bilthoven, The Netherlands; pierre.albert@rivm.nl; 2 School of Engineering, The University of Edinburgh, Edinburgh EH9 3JW, UK; 3 Usher Institute, Edinburgh Medical School, The University of Edinburgh, Edinburgh EH8 9YL, UK; s.luz@ed.ac.uk

**Keywords:** ambient intelligence, human-behaviour tracking, multimodal recording devices, privacy preservation, cooperative work, healthcare

## Abstract

The ubiquity of digital technology has facilitated detailed recording of human behaviour. Ambient technology has been used to capture behaviours in a broad range of applications ranging from healthcare and monitoring to assessment of cooperative work. However, existing systems often face challenges in terms of autonomy, usability, and privacy. This paper presents a portable, easy-to-use and privacy-preserving system for capturing behavioural signals unobtrusively in home or in office settings. The system focuses on the capture of audio, video, and depth imaging. It is based on a device built on a small-factor platform that incorporates ambient sensors which can be integrated with the audio and depth video hardware for multimodal behaviour tracking. The system can be accessed remotely and integrated into a network of sensors. Data are encrypted in real time to ensure safety and privacy. We illustrate uses of the device in two different settings, namely, a healthy-ageing IoT application, where the device is used in conjunction with a range of IoT sensors to monitor an older person’s mental well-being at home, and a healthcare communication quality assessment application, where the device is used to capture a patient–clinician interaction for consultation quality appraisal. CUSCO can automatically detect active speakers, extract acoustic features, record video and depth streams, and recognise emotions and cognitive impairment with promising accuracy.

## 1. Introduction

Multimodal recording of human actions, including voice, gestures, gaze, and gait, in a home or work environment can provide a rich source of information for the analysis of different aspects of behaviour and for unobtrusive monitoring of physical and mental health status [1,2]. However, data collection and analysis in such a scenario pose a number of challenges for researchers and designers of ambient intelligence (AmI) technology. These challenges include ethical as well as technological challenges. Ethical issues include obtaining informed consent, guaranteeing user privacy, avoiding biases in model development and inference, and other challenges [3]. Technical challenges include making the systems and devices usable and unobtrusive, portability across environments (e.g., home, assisted care, workplace, etc.) and application scenarios (e.g., continuous health monitoring, assisted living, collaboration and interaction assessment, etc.) [4], data management, and system performance, among other challenges, which are often intertwined with ethical and usability challenges. While AmI systems have been developed which address aspects of such challenges, these systems tend to be tailored to specific applications and often implement complex system architectures.

The users of such systems often feel uncomfortable about being recorded, and are not willing to share their audio-visual data related to their health conditions due to its high re-identification risk. We observed examples of this attitude among the SAAM project’s participants in our user studies [5]. We believe that the increasing incidence of high-profile data breach problems (https://www.bbc.co.uk/news/articles/cv2zy81j58yo—accessed on 20 February 2024) may contribute to such attitudes towards recording of personal information in identifiable form.

**Figure 1:**
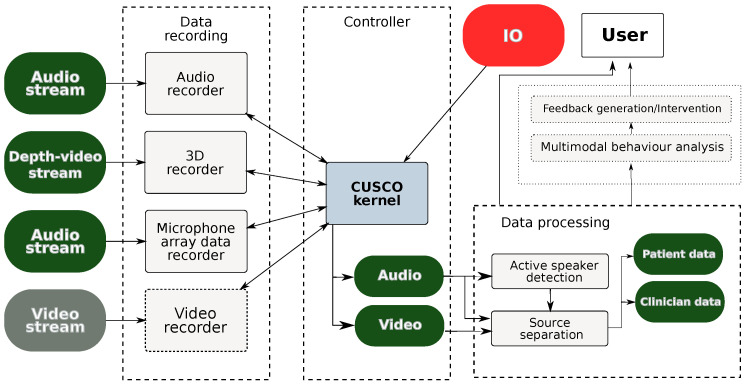
The CUSCO system architecture. The modules shown in dotted boxes are not presented in this paper.

We describe a minimalistic embedded system named CUSCO (custom secured recorder) which focuses on audio-visual monitoring based on a small, portable and general-purpose architecture as shown in Figure 1. CUSCO was developed in the context of a research project on interaction analytics for automatic assessment of communication quality in primary care (INCA), where it supported the automated analysis of communication patterns in medical interviews such as consultations and briefings. This setting involves patient–clinician dialogues in real-world environments where the researcher has little control over the environment and recording conditions. The device supports data collection of medical interviews by recording audio, video, and depth video streams with quality levels suitable for use with automatic speech and video processing techniques, while the recorded data pass through the first stage of feature extraction and are encrypted upon collection.

CUSCO comprises an 8 micro-electromechanical system (MEMS) microphone array, coupled with a stereoscopic depth camera with a field of view (FOV) of $87^{{}^{\circ}}\times 58^{{}^{\circ}}$ at the 0.2 m to 3 m range, and a video camera with a $90^{{}^{\circ}}$ FOV. It features a regular PC interface and remote access through secure shell (SSH), and can be integrated into a network of sensors. Data are encrypted in real time to ensure the recorded information cannot be compromised at any stage of the collection. In this paper, we illustrate uses of this device in two different settings, namely, a healthy-ageing AmI application, where the device is used in conjunction with a range of IoT sensors to monitor an older person’s mental well-being at home, and a healthcare communication quality assessment application, where the device is used to capture patient–clinician interaction.

The use of CUSCO in the AmI, healthy-ageing application took place in the context of an EU-funded project entitled Supporting Active Ageing Through Multimodal Coaching (SAAM). In the SAAM project, we employed IoT and AmI technologies to analyse the activities and health status of older people living on their own or in assisted care settings, and to provide them with personalised multimodal coaching. Such activities and status include mobility, sleep, social activity, air quality, cardiovascular health, diet, emotions, and cognitive status [5]. While most of these signals are tracked through specialised hardware, audio and speech are ubiquitous sources of data that can also be explored in these contexts. Speech quality and activity, in particular, closely reflect health and well-being. CUSCO has the ability to perform acoustic feature extraction for fusion with other sensor modalities, as well as stand-alone machine-learning-based mood assessment, depression detection, and emotion recognition, in resource-constrained settings. We trained machine learning models using publicly available data. Based on these models, upon detection of voice, CUSCO processes and saves emotion-, cognition-, and mood-related information.

As mentioned, CUSCO has also been employed to monitor clinician–patient communication in the context of the INCA project. The system was deployed in clinical settings (primary care practices, hospital rooms, etc.) to record medical consultations (audio, acoustic features, depth, voice activity and direction of arrival). It was operated by clinicians in their work location while the collected data had to be secure at all time. A crucial requirement was that the system had to have a minimal impact on their workflow and detect operational issues [6].

The system has been assessed in terms of how well it met its functional requirements, for usability, for suitability to the application contexts, and for its capabilities. The system allows the recording of human interactions in real-world settings, fulfilling all requirements defined in the application context. User evaluation and feedback have been collected to identify, document, and improve usability, as well as to address functional limitations. In addition to the INCA and SAAM projects, the CUSCO system has been used by two other research teams in the contexts of data collection and applications on cooperative support for people living with dementia. An evaluation of the out-of-the-box voice activity detection and diarisation capabilities of the system found its performance to be on par with state-of-the-art speech segmentation research software. The collected datasets have also been used to investigate markers of cognitive function and emotion in spontaneous speech [7].

The CUSCO system has real-world applications in care-home settings; the prototype is designed to address the privacy concerns of users as they are not willing to be recorded visually and also not willing to have a recording device in their homes. Based on their concerns, we specifically designed CUSCO to address their privacy concerns related to audio recording by extracting acoustic features using CUSCO and deleting the audio streams upon feature extraction. The prototype was presented in an outreach activity to around 60 elderly people at Red Cross Bulgaria. The feedback from the participants was very positive.

In terms of research requirements, using traditional devices such as video cameras and microphones will typically not provide researchers with data suitable for detailed behavioural analysis. These devices also pose privacy-breach and security risks if stolen. Laptops, tablets, and smartphones are not cost-efficient, and would introduce usability barriers for older people and cost busy professionals such as doctors precious time to set up. These limitations of current solutions regarding usability, operation, design, and security issues in care-home and office settings motivated the development of the CUSCO system.

This article discusses the requirements for the secure collection of dialogues containing sensitive information in real-world settings and their implementation through the CUSCO system. It illustrates the use of this system in the collection and analysis of medical interactions, presenting an analysis of audio-visual data for the exploration of automated appraisal of clinical communication, and in the monitoring of health and well-being in a home environment. The paper also discusses how CUSCO meets these requirements by providing a cost-effective device that combines data security and analytical capabilities on open hardware and an open-source software platform to allow researchers to easily reproduce and deploy the system. An evaluation and demonstration in practical applications is also presented.

## 2. Related Work

Technologies aimed at weaving computational resources into the fabric of everyday life have developed at an increasing pace over the past decades [4,8]. This trend towards ubiquitous and pervasive computing has opened new perspectives for applications related to human-behaviour monitoring. Assessment of group communication performance in real-world environments forms an important kind of such applications. These vary in context and settings, including early research on support for collaboration in meetings [9,10], speech analytics for group work in educational settings [11], support for healthcare work in hospital environments [12], as well as a wealth of applications in physical- and mental-health monitoring in living spaces [2]. These applications have been driven by advances in image, video, and speech processing and machine learning, which have enabled processing of multimodal signals for inference, adaptation, and decision making.

In terms of physical health and activity monitoring, a wide variety of sensors have been used in home environments, from Microsoft Kinect cameras to simple accelerometers. Often, a starting point for processing sensor data is to perform activity recognition. This allows the identification of routines, and departures from a baseline routine can be linked to various issues, for instance, dementia or depression [13]. Other applications include fall detection [14] and frailty classification [15,16]. Mental-health monitoring has also made extensive use of speech, though mostly such data have been collected in controlled rather than natural-living environments [17].

In addition to health monitoring, audio-visual data have been used frequently in the study of human behaviour and interactions. Data sets comprising recordings of clinician–patient dialogues have been used for assessing communication performance and the factors that affect it in medical consultations. Audio recordings have been used in the healthcare domain for content analysis, for assessing the effectiveness of face-to-face to telephone and video consultations [18,19], and for detecting Alzheimer’s dementia [20] through analysis of prosodic and paralinguistic features of spontaneous speech, among other applications. The combination of audio and visual data is often used in academic assessments of the performance of medical students and trainee general practitioners (GPs) in medical consultations, and in research on communication in healthcare, such as research on the impact of the introduction of electronic health records in GPs’ offices by monitoring the GP’s focus of attention [21].

Recent changes in the way data are considered have consequences on how corpora must be collected and handled. The introduction of the GDPR [22] in the European Union, for instance, imposes ethical and technical considerations for personal data in research, further constrained for medical data. However, research into the collection of data in AmI data has given less consideration to privacy and security issues than to issues such as feature extraction and machine learning [23,24]. Relatively few of the authors consider privacy in the context of low-resourced AmI in their work.

## 3. Requirements

For this work, the main objective was the secure collection of interactions for the exploration of automated processing and inference in group interaction and home-environment settings. This overarching goal implies several functional as well as non-functional (usability, safety, etc.) requirements, which we specify below.

### 3.1. Functional Requirements

The core functionality of a system aimed at assessing individual and group behaviour is the ability to record multimodal interactions in a manner that addresses the operational limitations of existing systems in terms of generating suitable data for analysis and meeting usability, privacy, and security requirements. A summary of the functional requirements we elicited for this work is provided in Table 1 and detailed below. These requirements were elicited in collaboration with clinicians [6] and home-care providers [5]. They are classified as design requirements, which relate to the overall design of the system, operation requirements, which relate to the system’s use, security requirements, and data requirements.

Given that speech is a reflection of many behavioural characteristics, the primary functional requirement of the system is the ability to capture the voices of the individuals involved. Therefore, the system must be capable of recording the voices of at least two participants, which may include a therapist and a patient, and possibly the patient’s companions, such as relatives or carers.

The second functional requirement is capturing the posture of a participant, such as a doctor, including their body posture, limb positions, and movements of the head. To ease the analysis of interactions, the system’s camera must be able to capture the participant’s position accurately. To the best of our knowledge, no existing integrated consultation recording system addresses this requirement satisfactorily.

Additionally, the inclusion of an integrated storage facility to accommodate the data gathered in professional settings, such as medical offices, constitutes the third functional requirement. It is important to allocate enough storage space to accommodate the entire data collection process. This means being able to record interactions over a week, which is estimated to require 20 h of interaction. This can be broken down into sixteen consultations of fifteen minutes each per day (two consultations per working hour) or twelve consultations of twenty minutes each.

The system must also be able to record interactions occurring in uncontrolled environments (fourth requirement). The system should have the capability to capture interactions in various environments, such as general practitioner offices, hospital rooms, conference rooms, and patient residences. The device must possess the capability to operate in many places, which may not be well equipped or optimised for capturing interactions. For instance, it may need to be placed wherever space is available, even in noisy environments.

Furthermore, it is necessary to ensure the security of both the data that are being gathered throughout the collecting process, and the access to those data. Consequently, it is essential to ensure the security of every access point to the system. The collected data must be safely kept and maintained in their secure state throughout the process of retrieval and transfer to their ultimate storage location by a researcher. Data protection is implemented by several technological and data management measures. Although the proposed system primarily emphasises the technological measures, the processing and administration of the obtained data, nevertheless, have a significant impact on the system’s functional requirements. This is particularly evident at each stage prior to the data being transferred to the ultimate storage location.

The sixth and final functional requirement is the need to record the interaction so as to simplify the examination of automated manipulation of the unprocessed data and their subsequent manipulation. The pre-processing and processing quality of data are contingent upon the quality of the recorded signal. In applications such as speaker diarisation, the presence of noise in a recording poses a challenge in distinguishing between speech and silence, and identifying the speech source. It is, thus, necessary to guarantee that the recorded data are of the appropriate kind, range, and quality to support and simplify automated processing.

### 3.2. Non-Functional Requirements

External viewpoints considerably enhance the value of non-functional requirements. Therefore, our first non-functional criteria were determined by the constraints imposed on the system by the ethical standards of the Irish College of General Practitioners (ICGP) and the SAAM project. To better understand these constraints, we held meetings with representatives from the ICGP and professionals to address issues and explore possible solutions. These meetings included presenting and discussing the challenges, receiving input on their acceptability, and gaining a better understanding of expectations, particularly around the usage of recording devices by GPs and in assisted-living facilities. Supplementary non-functional requirements were established through prototype development and use, supplemented by discussions with GPs and researchers participating in the projects. This included meetings, interviews, and informal interactions. Additionally, practical experience and comments were collected throughout the pilot sessions.

The non-functional requirements are shown in Table 2 and detailed below.

In the INCA project, the system was used mostly in professional settings, namely, in clinics and hospitals, by doctors or during patient studies. Hence, it is essential that the system does not cause disruption to the user’s daily activities. That is, it is necessary for it to be inconspicuous and easily incorporated into the healthcare workflow so as not to interfere with the work of the clinician or distract the patient. Unobtrusivity is, therefore, our first non-functional requirement. An innovative tool that required substantial effort on the part of the user would be promptly disregarded, resulting in increased attrition rates. This requirement implies that the user interface have a low overhead and be easy to use, resulting in a reduced likelihood of errors.

Errors often occur with human processes. Anticipating and addressing any issues that may arise during the device’s setup and use is essential. Several errors may be anticipated and verified, particularly with the correct hardware setup of the device (disconnected peripherals, cable misplacement) and its state (powered off/ready for operation/recording).

The equipment will be used by users with little familiarity with recording devices. Therefore, the professional (e.g., clinician) who operates the system, and the researcher who retrieves the data, should not be required to have an extensive understanding of the system. The operation of the system, including setup, configuration, and data retrieval, should be straightforward, unambiguous, and well documented.

The device will be relocated often to facilitate the activities of doctors and data-collecting partners in real-world, non-experimental settings. Therefore, it is crucial to prioritise robustness and ease of deployment as non-functional requirements, as these will also minimise potential disruption to clinical work.

Privacy concerns impose additional demands. To ensure the privacy of users as well as non-participating patients, it is necessary for the device to cease recording automatically if it is not manually turned off after a certain period of time. Determining when the recorded clinical encounter has ended poses significant challenges in the anticipated use cases. Designing an automated method for stopping the recording is non-trivial. One could consider stopping after a silence-interval threshold is exceeded, but in practice, extended periods of silence may occur during medical consultations. Alternatively, the system might allow the user to set a pre-defined recording period based on the anticipated period. If the recording is continuously active, the device should restrict excessive recording. Although the subsequent interview that is not part of the planned recording will be erased during data retrieval, the device should minimise the amount of this interaction that is actually captured.

Within a consultation environment, the system should enable the investigation of the actions performed by health professionals. However, it is imperative to protect the patient’s identity in the recording. If a patient happens to pass by the camera’s field of view while a medical consultation is being recorded, it is important to ensure that their identity remains confidential, especially if the room’s layout makes it possible for this to occur. Similar requirements apply to home-care use cases.

Internet connectivity in rural places, and even in certain regions inside major cities, is of insufficient quality to support the efficient transmission of large volumes of data. Excluding the possibility of transmitting the recorded data over the Internet in certain cases, it is necessary to provide for both storage and retrieval of the recorded data. The system should, therefore, facilitate access to the recorded interaction, allowing for easy retrieval of data by a researcher who may not have expertise in the field. This process should not disrupt the ongoing collection of data; it should not necessitate the use of a new device or require extensive processing time before the device can be used again.

There are other technical factors to consider. The device may experience adverse electromagnetic interference (EMI) in many contexts, particularly when medical equipment is present. This interference might cause noise that affects audio capture. A solution is required to alleviate this problem. This requirement was established subsequent to the discovery that EMI had affected the recordings in our early prototypes.

Additionally, arrangements should be put in place to accommodate further development and extension of the system. The system should use interchangeable components that can be easily substituted with others, in accordance with the life cycles of industrial goods, while minimising any unnecessary overhead such as extensive reconfiguration. Additionally, it should accommodate new requirements while keeping upgrade costs to a minimum.

Finally, the system development took place in the INCA and SAAM projects, two research projects in which the deployment of multiple units was foreseen. In addition, both software and hardware have been used to collect spoken interactions with people with dementia as part of a PhD project [25]. Therefore, cost efficiency is an important factor.

## 4. Materials and Methods

### 4.1. Hardware

To support the collection of interaction data in real-life settings, such as real consultations in a GP’s practice or training sessions in a medical school, where recording is but an ancillary activity, a system must have the smallest impact possible on the workflow. As such, systems that have been used so far are either complex to set up—computer(s) with plugged-in devices—which can be disruptive or would require the presence of a researcher. In most cases, as discussed above, such setups are not secure.

This gap between practical requirements and existing systems led us to investigate the creation of a small, simple, self-contained device that could meet the different requirements identified in the previous section for the collection of face-to-face interaction data containing private and potentially sensitive information.

The integration of all of the requirements mentioned above entails the implementation of solutions that may contradict each other and impose restrictions on system functionality. Consequently, our design choices were informed by the discoveries in the existing literature, prevailing methods, and stakeholder feedback.

The proposed system provides a specialised platform designed specifically for the envisioned use cases: it is secure, capable of gathering audio and video data, easy to implement, user-friendly, and adaptable. The CUSCO system incorporates two established methods for securely storing data: it includes a self-contained computer presented to the user as a physical box with a minimalist interface.

**Figure 2:**
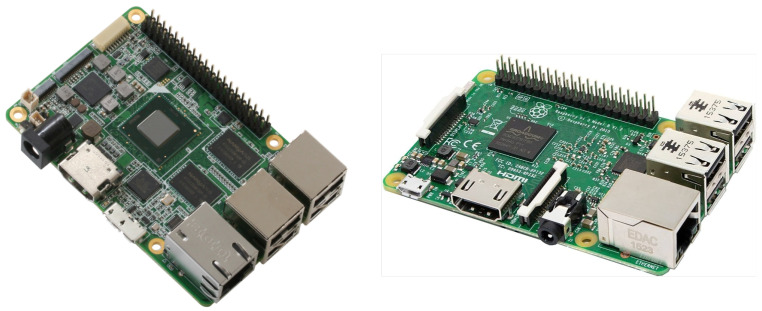
Intel UpBoard (**left**) and Raspberry Pi 3B (**right**).

**Figure 3:**
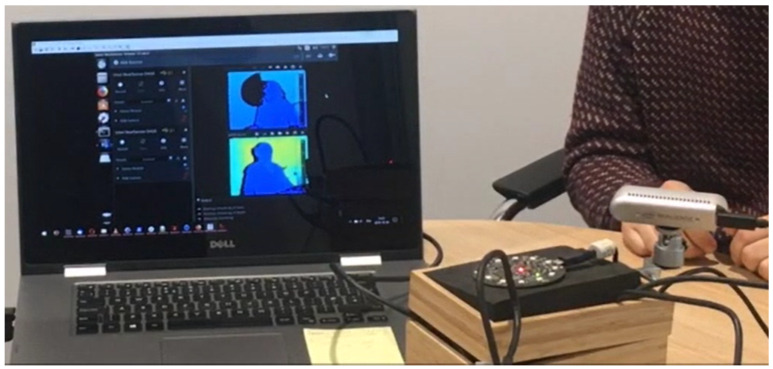
Cusco demo for recording videos in an office setting.

The system is based on a small form-factor computer measuring 2 cm in thickness and resembling a credit card, as shown in Figure 2. Different versions of the system used either an Intel UpBoard, built around a Quad Core Atom X5-8350 CPU (https://up-board.org/up/specifications/ accessed on 20 February 2024), or a Raspberry Pi 3 (model B), which is based on a Quad Core 1.2 GHz Broadcom BCM2837 64-bit CPU (https://www.raspberrypi.com/products/raspberry-pi-3-model-b/ accessed on 20 February 2024). The computer is enclosed inside a small box and contains functional hardware such as cables and boards, as shown in Figure 3. The computer boards use either the x86-64 or the ARM A32 instruction set. These architectures are very prevalent in servers and personal computers, guaranteeing seamless compatibility with the majority of software designed for these systems.

We employ a microphone array and a 3D depth sensor to capture audio and video streams with sufficient quality for the application of automated processing methods, based on findings from earlier experiments. The device is compact and conveniently suitable for placement on tables or desktops. The hardware and software provide modularity, which allows for the integration of new research and data-gathering requirements. Any element may be substituted without requiring any modifications to the original code. Every sensor is encapsulated inside a separate software module, allowing for individual configuration and replacement without necessitating modifications to any other modules.

The programming language used for the implementation of the global networking, controllers, and modules was Python, which is a widely used programming language [26]. Python operates at a high degree of abstraction, minimising the challenges associated with hardware and memory management. However, some components could not be controlled with Python or had to rely on other dependencies, and therefore, had to be developed in other programming languages. The 3D stream/video recording thread, for instance, was implemented in C++. The initial setup of the operating system (OS) required the use of internal OS tools and programming languages. This included tasks such as enabling the encrypted hard drive and initiating the modules and controller.

The overall specifications of our proposed solution, the CUSCO device and accompanying software, can be summarised as follows:

- Open-source license: GPLv3;
- Cost of hardware: approximately GBP 300;
- Hardware, source file repository: Source code at osf.io;
- Software repository: gitlab.

The device was built in two versions: an audio-only version that was designed for the SAAM assisted-living setting, where the device provides a speech-based interface to other AmI components of SAAM and monitors the user’s speech for mood and cognitive assessment; and an audio–video version (depth video, infrared, and conventional high-resolution video) equipped with a minimalist user interface for recording of medical encounters and consultations in the INCA project. In the following sections, we describe the software modules of the system.

### 4.2. Data Storage

The primary factor to be taken into account for data storage (FR3, Table 1) is dimensioning. The computer boards we employed have a restricted amount of internal storage memory, which is insufficient for storing large datasets. Simulations and testing were conducted to evaluate the requirements for storage space, as shown in Table 3. We found that a typical session would need around 15 GiB of storage to capture voice, direction of arrival, and 3D stream data. Hence, it was necessary to use specialised storage. The storage capacity had to be enough for data gathering activities that lasted over a few days. Storage allocation was required for a period of one week, namely, for five business days. Each day consists of 10 sessions, resulting in a total storage need of around 750 GiB. The closest size range for commercial storage devices is 1 terabyte (equivalent to 931 gigabytes), with a margin of 24% (12 sessions). The selection of an external hard-disk drive was based on cost considerations.

The second factor to take into account for data storage is the logical aspect, namely, the software component. When storing encrypted data, one must consider the encryption technology used and the format of the storage space, known as the file system. The need for interoperability posed a challenge in choosing a file system, since there is no universally compatible file system available. The Linux ext3 file system was chosen among the available choices due to its widespread use on this platform and the availability of several tools that can access this format on other platforms [27].

### 4.3. Data Security

CUSCO offers a significant advantage in terms of data security. To ensure the security of a system, it is necessary to adhere to standard protocols and guidelines. The National Cyber Security Centre (NCSC) has set out design standards for high assurance products which encompass six core principles. While the CUSCO system is not designed to operate in high-threat environments, the NCSC guidelines provide a strong foundation for the creation of a safe system. In brief, the system deals with these data security principles in the following manner:

**Trusted sources:** “Products should be sourced from trusted suppliers with proven high-threat-domain knowledge”. Each security aspect of the CUSCO device relies on a specialised tool developed and provided by a team or firm devoted to security. This ensures that the tool is supported and monitored, and that we have a thorough understanding of the associated environment.

**Good practice:** “Developers’ processes should demonstrably meet all accepted good practice”. There are four important prerequisites in this regard: (1) using a configuration management system, (2) using an issue tracker for addressing identified flaws, (3) establishing a flaw-reporting mechanism for external reviewers, and (4) conducting thorough testing of the product. In relation to (1–3), our chosen method for configuration management included self-assessment, software version control, and documentation (for both software and hardware). In relation to (4), a more minimalistic approach was adopted: security elements were designed and implemented via discussions with INCA team members and other researchers, and the system underwent testing by other researchers.

**Documentation of security functionality:** “Products should have clearly defined, specific security functionality, with limitations identified”. The fundamental basis for the security management of data storage and device access in the CUSCO system relies on the use of established and well-documented third-party solutions, which provide a clear understanding of their capabilities and limits. Three distinct stages are relevant to ensure data security: data collection, data storage, and data retrieval and transportation. The data must be maintained independently from the collection for ultimate storage and usage, such as on a data server. This is necessary since the needs for storage, sharing, processing, and other factors may vary. The data must be safeguarded throughout the three stages, with little human involvement, to prevent errors. During data collection, it is essential to encrypt the data in real time as it is recorded. In the event that the device is hacked or stolen during recording, loss of power will hinder the device’s ability to internally identify and complete ongoing procedures. This vulnerability was mitigated by the use of on-the-fly encryption. The data encryption algorithm used was AES. A cryptographic hash function (CHF) complemented the encryption, mapping data of different sizes into fixed-size blocks. AES was used in conjunction with the Secure Hash Algorithm 512 (SHA-512). AES is a symmetric block cipher algorithm which is capable of handling data blocks of 128 bits, using keys with lengths of 128, 192, and 256 bits. It employs the same key (cipher) to encode and decode data blocks. Encryption involves substitution, shifting, mixing and further encryption. SHA-512 is derived from the SHA-2 (Secure Hash Algorithm 2) series of hash algorithms, which perform a one-way mapping of an arbitrary binary string to a smaller string of fixed length. The SHA algorithms were introduced by the NIST [28]. AES and CHF are currently regarded as secure, since there are no known attacks that can be carried out using computational methods.

**Support of assessment:** “Products should support systematic, independent and evidence-based assessment of claimed security functionality”. This was accomplished via the use of open-source security measures. By releasing the source code under an open-source license and making it publicly available external reviewers are able to conduct thorough code reviews. Regarding hardware, the publication and documentation of the device provide comparable safeguards.

**Operation:** “Products should always operate as intended”. The CUSCO documentation provides a detailed description of the device’s features and mode of operation. There are no undocumented features. The device’s minimalistic user interface restricts its use to the available inputs (such as switch on, switch off, power on, power off), thus safeguarding the device from any errors in operation. The recorded data are encrypted during the data-frame recording to provide comprehensive security against failure and interference.

**Trusted state:** “Products should always be in a trusted state”. It is necessary to monitor the integrity of the components that make up or are connected to the device. This is partially ensured by an internal configuration check, which verifies the existence and functionality of the storage device, as well as the presence of the anticipated sensors for a certain configuration. This check is performed both at start-up and at regular intervals throughout the operation of the device. During operation, the internal status of each module is monitored, and if any abnormalities are detected in the sensors or storage space, the device’s recording capability is temporarily halted until the condition returns to normal. Nevertheless, peripheral devices undergo no security checks, allowing for the possibility of any device being replaced with another device of the same type (sensors) or configuration (storage device encrypted with the same encryption key). Although there is a potential for attacks employing compromised devices, we deem this risk to be acceptable owing to the need for very advanced equipment and expertise to execute such attacks.

The above principles ensure that user information stored in the device enjoys acceptable levels of security. Once these data are extracted from the device, best practice suggests that they are kept encrypted and physically secure. However, we have implemented additional measures to preserve user privacy should data need to be shared. These measures include feature extraction techniques that preserve content-privacy and speech disguising. These methods are described in detail in Section 4.4.2 and Section 4.4.5.

### 4.4. Audio Capture Module

The audio capture module is based on a Matrix Voice™ microphone array board. This platform consists of a field-programmable gate array (FPGA) Xilinx Spartan 6 XC6SLX9 chip, an array of 8 MEMS microphones, 18 RGBW LEDS that may be used to indicate the audio detection and its direction of arrival, RAM 64 MByte 132 MHz SDRAM, a FLASH 64 Mbit memory chip, a WiFi 2.4 GHz 802.11 bgn module, and an LE-Microcontroller Tensilica Xtensa dual-core 32-bit LX6. The FPGA is an integrated circuit that can be programmed. It allows some audio pre-processing, such as the detection of streams arriving from different directions, which can provide input to a speaker diarisation module (Section 4.4.1). In some settings of the SAAM project we used the Matrix Creator™ instead of the simpler Voice model. Matrix Creator™ encompasses other sensors that were used in other parts of the SAAM system which are not described in this paper.

#### 4.4.1. Active Speaker Recognition Module

We developed an active speaker recognition module using the capabilities of the microphone array, open-source software, and a combination of both. This provides voice activity detection (VAD), speech turns segmentation, and speaker diarisation.

We broke down the assessment of this module of the system between the VAD and speaker diarisation. This provides an insight into the potential of the system for each of these tasks, which can be used to support data pre-processing for manual analysis (e.g., in qualitative analysis of medical consultations) for fully automatic processing.

Speaker diarisation is the task of segmenting an audio stream into a sequence of speech turns and assigning a speaker identifier to each of these segments [29]. The diarisation error rate (DER) is the most commonly used metric in the evaluation of speaker diarisation systems. DER is defined as the proportion of speaker time that is mislabelled in relation to an optimal one-to-one mapping of reference speaker labels to system output speaker labels [30].

We also computed the $P_{k}$ metric [31] and the WindowDiff (WD) metric [32] to provide a baseline for VAD and speech segmentation. Originally developed for text segmentation, these metrics are also used to evaluate the segmentation of spoken interactions [33], but do not process assigned speakers.

We define $P_{k}$ as the probability that two turns occurring *k* turns apart and picked otherwise randomly from the dataset are incorrectly identified by the algorithm as belonging to the same or to different speakers. This is formally stated in Equation (1), where *r* and *h* denote the reference and hypothesis segmentation, respectively. $D_{k}$ stands for a distribution with probability fixed at a distance *k* (chosen to be half the average segment size, in number of turns), a(i,j) returns 1 if *i* and *j* belong to the same speaker and 0 otherwise, and δ returns 1 if its two arguments are equal and 0 otherwise (Kronecker delta). This results in an increment if boundaries are assigned inconsistently within a segment.

(1) $$P_{k}\left(r,h\right)=\sum_{1\leq i\leq j\leq N}D_{k}\left(i,j\right)\left[1-\delta \left(a\left(r_{i},r_{j}\right),a\left(h_{i},h_{j}\right)\right)\right]$$

WD is a measure of the discrepancies between a reference and a hypothesis. It is calculated by moving a window of length *k* segments over the recorded audio and tallying the number of disagreements. The error score is calculated by considering both the anticipated number of boundaries by the algorithm and the actual number of boundaries in the reference. The score is computed according to Equation (2), where *N* represents the count of sub-segments with a size of *k*, as before, and b(i,j) denotes the number of speaker boundaries between turns *i* and *j*.

(2) $$\mathrm{WD}\left(r,h\right)=\frac{\sum_{i=1}^{N-k}\left[1-\delta \left(b\left(r_{i},r_{i+k}\right),b\left(h_{i},h_{i+k}\right)\right)\right]}{N-k}$$

#### 4.4.2. Acoustic Feature Sets

Three features sets are extracted by the system, which have been widely used for emotion recognition [34,35], eating conditions recognition [36], and cognitive impairment [37]. These sets are (a) emobase, which is an acoustic feature set containing mel-frequency cepstral coefficients (MFCCs), voice quality, fundamental frequency (F0), F0 envelope, LSP, and intensity features, along with their first- and second-order derivatives, along with many statistical functionals, resulting in a total of 988 features for every speech utterance; (b) ComParE [38], which comprises energy, spectral, MFCC, and voicing related low-level descriptors (LLDs), including logarithmic harmonic-to-noise ratio, voice quality features, Viterbi smoothing for F0, spectral harmonicity, and psychoacoustic spectral sharpness, for a total of 6373 acoustic features per utterance; and (c) eGeMAPS [35] which contains the F0 semitone, loudness, spectral flux, MFCCs, jitter, shimmer, F1, F2, F3, alpha ratio, Hammarberg index, and slope V0 features, and their functionals, for a total of 88 features per utterance.

#### 4.4.3. Acoustic Feature Extraction and Integration of Modules

**Figure 4:**
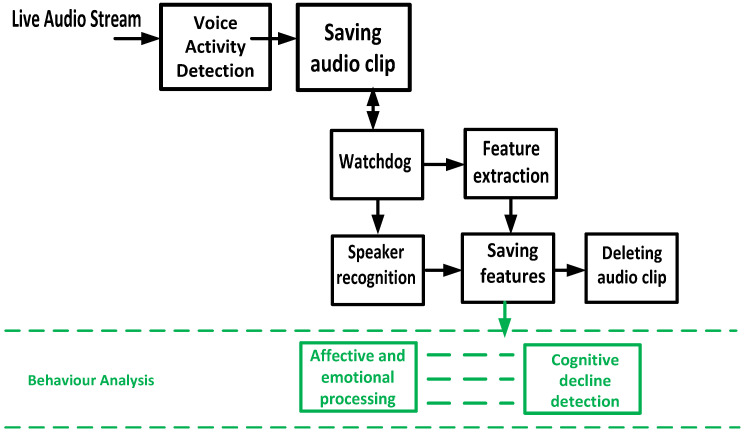
Feature extraction pipeline.

The method of feature extraction is shown in Figure 4. The Auditok library [39] is used for real-time speech chunk recognition by analysing the energy of the audio signal. The detected chunks are then stored as pulse-code modulation (PCM) streams. The input for the user identification module and audio feature extraction library [38] consists of these streams. The user recognition module analyses the input to differentiate between streams that include the voice of the target user and other noises, such as additional speech or background noise. The latter individuals are disregarded in order to provide further protection for the privacy of those who have not given consent to participate in the conversation in question. After extracting the privacy-preserving auditory information, the PCM streams are promptly erased. The software used in these processes can be accessed via our git repository (git@git.ecdf.ed.ac.uk:fhaider/saam-av-capturing-system.git). The extracted features are then used to detect emotion, and for cognitive impairment detection.

#### 4.4.4. Emotion Recognition Module

The CUSCO system includes an emotion recognition algorithm that we designed particularly for situations when it is important to reduce memory use and computational demands. One way to achieve this objective is by decreasing the number of features used for machine learning model inference. Therefore, we assessed three advanced feature selection techniques: infinite latent feature selection (ILFS), ReliefF, and generalised Fisher score. We then compared these approaches to our newly developed feature selection approach called “active feature selection” (AFS). The assessment was conducted on three emotion-identification datasets (EmoDB, SAVEE, and EMOVO) using two established acoustic paralinguistic feature sets (namely, eGeMAPs and emobase). The findings show that the suggested technique can produce comparable or superior accuracy compared to other current methods, even when using significantly smaller subsets of features instead of the complete feature, set as shown in Table 4.

#### 4.4.5. Speech-Disguising Module

In order to meet privacy regulations, we have included an algorithm that records altered voice signals, thereby safeguarding the user’s identity via the use of pitch-shifting techniques [40]. The pitch-shifting techniques alter the speech signal by modifying its pitch but maintaining the duration of the speech signal. The covertly captured speech can be used to train machine learning models for the purpose of emotion identification. To achieve this objective, we assessed the acoustic characteristics derived from both undisguised and disguised speech in order to perform an affect recognition test. This was achieved by using six distinct machine learning classification techniques. Additionally, the study showcased the outcomes of applying transfer learning techniques from non-disguised speech to disguised speech. We found certain auditory properties that were unaffected by the pitch-shifting algorithm. Furthermore, we assessed these features for their ability to recognise emotions. Our findings indicate that the non-disguised speech signal achieves the highest unweighted average recall (UAR) at 80.01%. However, the disguised speech signal only slightly reduces performance, reaching 76.29%. Transferring knowledge from non-disguised to disguised speech leads to a decrease in the UAR from 76.29% to 65.13%. Nevertheless, feature selection enhances the UAR (68.32%).

#### 4.4.6. Cognitive Impairment Detection Module

We evaluated the extended Geneva minimalistic acoustic parameter set (eGeMAPS), the emobase feature set, the ComParE 2013 feature set, and the newly developed Multi-Resolution Cochleagram (MRCG) features [34]. In addition, we propose a novel technique called active data representation (ADR) for extracting features for detection of Alzheimer’s dementia speech in low-resource environments [37]. The results indicate that classification models relying just on acoustic speech characteristics extracted using our ADR technique can achieve accuracy levels similar to those reached by models including more advanced language features. The analysis of the findings indicates that each feature set provides unique information that is not captured by any other feature set. We demonstrate that the eGeMAPS feature set exhibits somewhat superior accuracy compared to other individual feature sets (71.34%). However, the amalgamation of feature sets, known as “hard fusion”, enhances the accuracy to 78.70%. A comparison with other methods is shown in Table 5.

### 4.5. Depth and Video Stream Capturing Module

Depth (3D) video tracking is often used for the purpose of monitoring and analysing body posture ([45,46]), gestures ([47]), and head poses ([48]). The Intel RealSense, a prototype 3D sensor designed for low-processing-power platforms, was chosen over the Microsoft Kinect camera for these purposes. We conducted experiments using two distinct models, as shown in Table 6. Ultimately, we selected the D435 model. The incorporation of upgrades to the CUSCO system was made possible by the introduction of the final versions of this sensor. The 3D sensor has a resolution of 1280 × 720 and operates at a variable frame rate ranging from 6 to 90 frames per second (fps). However, there are restrictions on the possible combinations of resolution and frame rate (Datasheet for the camera is available at https://www.intelrealsense.com/wp-content/uploads/2020/06/Intel-RealSense-D400-Series-Datasheet-June-2020.pdf, accessed on 20 February 2024).

Acquiring the sensor’s data at its highest level of detail and speed (1280 × 720 at 30 fps or 848 × 480 at 90 fps) requires a significant amount of resources, both in terms of computing power during recording and in terms of storage space.

Recording a 3D stream enables capturing the whole body position of a single person, including the torso, limbs, and head. The purpose of capturing the 3D stream in CUSCO is to enable the monitoring of large-scale bodily characteristics, such as the skeleton, upper body, and head position. The tracking of intricate details such as the hand, fingers, or movements may be achieved with a higher frame rate. This allows for the examination of gestures within shorter time intervals, such as 11 milliseconds at 90 frames per second compared to 33 milliseconds at 30 frames per second. However, these capabilities were not considered necessary for the envisaged use scenarios.

Consequently, by decreasing the recorded resolution and frame rate, the use of resources was minimised while maintaining the ability to analyse data. The stream was, therefore, recorded at a resolution of 640 × 480 and a frame rate of 30 frames per second.

A dedicated module was supplied to capture video camera feeds to allow the extraction of detailed visual data such as facial features, eyebrows, and gaze direction.

## 5. The System in Use

### 5.1. Data Collection

The first prototype of the CUSCO system was used for two patient–clinician data collection tasks in the INCA project, and a different data collection task in the SAAM project. In both cases, we were able to demonstrate the system’s ability to integrate and fuse different data sources, including speech, video, and 3D point clouds. As the system was mounted on a single board, these data streams could be seamlessly time-aligned. Synchronisation profiles can also be generated for analysis of the different streams on existing software, such as ELAN [49]. The data collected through CUSCO in the SAAM project can be made available to researchers upon reasonable request. The consultations recorded in the INCA project constitute private patient information, and therefore, cannot be shared with the research community.

In brief, the following capabilities and issues were identified:

- The system was able to handle the full data collection for each event. The system allowed the recording of dyadic interactions in real-world settings, fulfilling every requirement set.
- Limitations were identified for recording scenarios extending the use beyond the planned setting, e.g., in large hospital bedrooms instead of smaller GPs and meeting rooms. Recording distances past the limits of the microphone array (3 m) resulted in a low volume of recording, leading to difficulties in the automated analysis of the data.
- Similarly, when speakers are located in close azimuths (<45° angles) from the device, voice activity detection cannot distinguish between both signals and becomes irrelevant for diarisation.
- During the second data collection, the cable of the camera was unplugged while changing the location of the device, leading to a loss of 3D stream recordings for a few sessions. An update of the internal status monitoring system of the 3D recorder module and other sensors was developed to help users of the system to identify the issue.

#### 5.1.1. Collection of User Data in Assisted-Living Settings

The above-described method was used to gather privacy-oriented features (such as eGeMAPs) from audio recordings in the Bulgarian language, in addition to self-reported mood, as part of the SAAM project. In this pilot investigation, privacy-related characteristics were retrieved from 16 recordings involving four people. The privacy-related characteristics are the acoustic representation of speech that guarantees the spoken content privacy and recovery of original speech through synthesis. In the regression analysis, we computed the mean of the privacy-based features, which were retrieved for each speech segment, throughout the whole audio recording. The regression study was conducted using linear and random forest regression techniques in a leave-one-(recording)-out cross-validation framework (i.e., 15 recordings were used for training and one for validation in 16-fold cross-validation), using MATLAB [50]. For assessment reasons, we used the concordance correlation coefficient (CCC) and the Pearson correlation coefficient (r). Our analysis revealed that linear regression had superior results compared to random forest in terms of Pearson correlation scores. Specifically, the correlation coefficient for linear regression was determined to be 0.338, while for random forest it was 0.256. Nevertheless, random forests yielded the highest CCC (0.237) when predicting pleasure. Random forest outperformed linear regression in predicting the arousal score, as shown in Table 7.

#### 5.1.2. Collection of Clinician–Patient Interaction Data

The system was used to collect three datasets.

The first dataset was collected as a pilot during the development of the system. It consisted of 10 sessions between two persons set in office environments (small and large meeting rooms, open space). Recordings were performed using the v1 system.

The second dataset was collected at an Irish GP practice and consisted of medical consultations between two different general practitioners and seven different patients. Recordings were performed using the v1 system.

The third dataset was collected at an Irish hospital, recording student training sessions with simulated patients. The corpus was collected using the v2 system, and consisted of 53 dyadic sessions (each with one nurse and one simulated patient).

The collection of two additional corpora with patients suffering from dementia are in progress. The first corpus consists of recordings of interactions between a patient and a researcher performing a map task [7], in a collaborative exercise to elicit spontaneous spatial navigation dialogues. The second corpus consists of interaction between a patient and a healthy participant, with the aim to collect casual conversations alongside facial features while performing the task [25]. This last data collection involves the use of two paired CUSCO devices, recording two microphones (one microphone array, one high-quality table microphone) and two video streams. Pilot recordings have already been performed for this setup.

### 5.2. System Evaluation

The evaluation of audio recordings focused on their capacity to enhance speech processing. The chosen metrics assess typical early stages in a speech processing pipeline, namely, speech segmentation and diarisation. Speech segmentation refers to the identification of speech segments in audio, and diarisation is the process of determining who talks at what time. These procedures are essential for further analysis, including both verbal analysis such as automatic speech recognition, and non-verbal analysis such as prosody.

We calculated the aforementioned $P_{k}$ and WD measures in order to provide a reference point for speech segmentation.

The system’s performance was evaluated on a small collection of meetings (total duration of 20 min). Two voice tokenisers were used for the purpose of comparison. Auditok, available at https://github.com/amsehili/auditok (accessed on 20 February 2024), is a tokenization tool that uses energy-based methods to conduct segmentation. However, it does not provide speaker labelling. LIUM [52] is a speaker diarisation tool that utilises the ALIZÉ speaker recognition library. The findings are shown in Table 8. The out-of-the-box voice activity detection performance is comparable to the latest technologies used in speech segmentation research. The use of VAD to enhance the outcomes has shown varying results when combining the output of speech segmentation software with the output from the integrated detection. The $P_{K}$ shows improvement, but the WD worsens with both the LIUM (−3.47/+14.87) and Auditok (−6.05/+12.33) tools. The performance of DER was likewise worse in terms of combined segmentation.

## 6. Discussion

A search of the literature on studies in medical teams and clinician–patient communication including a data collection component identified a distinct absence of consideration for many of the requirements presented in the Section 3. No secure or privacy-by-design systems were identified in any of the studies found involving data collection [53,54,55]. In cases where security is addressed, it is usually not directed at the recording devices or the security of the data stored in it, but at security of web servers where these data are stored, down in the data management pipeline [56], with some systems adopting questionable privacy practices such as passing patient voice data onto web-based services for transcription and often for storage [57]. Nor are security and privacy requirements usually adhered to in ad hoc recordings often made in clinical practice, either by the clinician or (less commonly) by the patient [58], with unclear legal implications [59]. This inattention to security and privacy requirements at the early stages of data collection was observed not only in many studies in the area of patient–clinician communication, but also in different corpora of clinical encounters, where data collection is usually performed with simple audio or video recorders, or microphones connected to one or more computers. The One in a Million archive [60], for instance, was collected using conventional cameras.

Non-functional requirements benefit greatly from external perspectives. As the original non-functional criteria were established based on the restrictions derived from the ethical standards of the ICGP, interactions and meetings with members of the ICGP helped us understand expectations regarding the use of CUSCO by GPs. During these meetings, several options were discussed, and feedback was offered on their acceptability. Supplementary non-functional requirements were collected via discussions, interviews, and informal interactions with general practitioners and academics who were engaged in the project. Additional insights and input of a more hands-on and pragmatic character were also obtained throughout the pilot sessions.

The pilot sessions included consultations conducted in an Irish general practice by a senior GP and a junior doctor. This collection was conducted as a pilot to gather data on medical interactions with the CUSCO device. A researcher initially managed the data collection, including setting up the device, providing explanations, and retrieving the data. The doctors presented the patients with consent papers that had been authorised by the ICGP. The doctors’ consents were obtained using an analogous form. The device was first deployed at the start of each collecting day, and thereafter, controlled by the doctors’ themselves throughout the recorded sessions. Subsequently, they were questioned about their use of the device and its effect on their daily activities subsequent to the first trial sessions. The suggestions and comments provided were used to incorporate modifications and to rectify the problems identified.

The system has garnered the interest of other researchers and has been used for various other data collection projects, such as for gathering conversations between individuals with Alzheimer’s disease and healthy volunteers. Requirements for this type of dialogue differed from the requirements of the INCA or SAAM projects. These new requirements included the need to capture a video of both participants at a resolution of 1280 × 720, as well as the usage of an extra high quality microphone. The system’s modularity facilitated easy adaption by coupling two devices, a main device and a secondary device, and synchronising their functioning based on the implemented modular architecture. A more rigorous evaluation of the audio and video quality across use cases, speech processing tasks, formal usability evaluation, and further comparison with existing systems would complement the current evaluation.

## 7. Conclusions

This article describes an embodied system for audio-visual data collection with an ability to automatically detect the active speaker, emotions, and cognitive impairment. The CUSCO system addresses a complex set of requirements that characterise the collection of audio-visual data in real-world settings, particularly in situations involving patients and vulnerable participants who have stringent data security and privacy requirements.

A current limitation of the system is that it only uses audio information for active speaker detection, emotion recognition, and cognitive impairment detection. This issue will be addressed in the future by incorporating visual information [61] to the active speaker detection algorithm. Currently, the system is trained on the English language and the performance of system modules related to cognitive impairment detection could be reduced when applied to other languages. The emotion recognition module is trained using Italian, English, and German language corpora.

Planned future work also includes the development of automatic detection and analysis of gestures and affective behaviour components for CUSCO using audio-visual information. Furthermore, in the patient–clinician communication setting, we also intend to explore methods to capture the general posture of the clinician. A clinicians’ posture and movements are important elements in developing rapport and responding to patients’ emotions [62]. These elements may also be related to accidental interruptions in dialogues and disclosure of information [63], which are active topics of research in this field.

## Figures and Tables

**Table 1:** Functional requirements of the CUSCO system.

ID	Type	Description
FR1	Design	Record interactions: speech of (at least) two participants.
FR2	Design	Record interactions: general posture of one participant.
FR3	Design	Storage space for multiple sessions.
FR4	Operation	Record interactions in typical settings for medical consultations.
FR5	Security	Securely record and store the data, and any access to it.
FR6	Data	Recorded data must allow automated processing in terms of quality and type.

**Table 2:** Non-functional requirements of the CUSCO system.

ID	Type	Description
NFR1	Usability	Unobtrusive.
NFR2	Usability	Ease of use by non-experts.
NFR3	Operation	Mitigate expected problems.
NFR4	Operation	Ease of operation by experimenters.
NFR5	Operation	Ease of deployment by experimenters.
NFR6	Design	Robustness.
NFR7	Security	Minimise breach of privacy if device is left recording.
NFR8	Security	Protect visual identity of patients.
NFR9	Design	On-site retrieval of the data.
NFR10	Design	Protection from EMI.
NFR11	Design	Upgradeable.
NFR12	Design	Modular.
NFR13	Design	Cost-efficient.

**Table 3:** Bitrate and storage requirements for the streams of each modality. Values are averaged over two sessions of 20 min.

Description	DoA (csv)	Audio (WAV)	Audio (FLAC)	Video (640 × 480)	Video (1280 × 720)
Bitrate (per second)	17 B	1536 KiB	189 KiB	11.09 MiB	40.11 MiB
Bitrate (per minute)	1 KiB	10.70 MiB	1.35 MiB	665.40 MiB	2.35 GiB
20 min session	20 KiB	214 MiB	27 MiB	13 GiB	47 GiB

**Table 4:** Best unweighted average recall (UAR (%)) of feature selection methods and number of selected features (numFeat) are reported. The best UAR (%) results for each feature set are given in bold. The unweighted arithmetic average for each feature selection method is also reported in the ‘average’ column. The basseline method uses the full feature set, i.e. no feature selection.

Dataset	EmoDB				EMOVO				SAVEE				
Feature Set	eGeMAPs		emobase		eGeMAPs		emobase		eGeMAPs		emobase		
	numFeat	UAR (%)	numFeat	UAR (%)	numFeat	UAR	numFeat	UAR (%)	numFeat	UAR (%)	numFeat	UAR (%)	Mean
Baseline	88	68.5	988	74.6	88	37.4	988	34.4	88	40.8	988	38.1	49.0
ILFS	74	**69.7**	685	**76.9**	28	38.1	113	34.7	86	42.0	574	38.8	46.9
ReliefF	88	68.5	666	75.3	20	37.8	348	**37.1**	82	41.4	72	39.3	49.9
Fisher	88	68.5	975	75.2	25	**41.0**	464	36.2	34	**42.4**	158	**42.4**	**51.0**
AFS	81	68.5	696	75.8	2	39.0	56	36.4	68	40.5	21	37.5	49.6

**Table 5:** Comparison with the state of the art.

Study	Accuracy	Modality	Fully Automatic
CUSCO	78.7%	acoustic	yes
Hernández et al. [41]	62.0%	acoustic	yes
Mirheidari et al. [42]	62.3%	text	yes (ASR)
Fraser et al. [43]	81.9%	text/acoustic	no (text)
Yancheva and Rudzicz [44]	80.0%	text/acoustic	no (text)
Hernández et al. [41]	68.0%	text	no
Mirheidari et al. [42]	75.6%	text	no

**Table 6:** Video and three-dimensional sensors specification. Resolution is measured in pixels, frame rate is measured in frames per second (fps), range is measured in meters, and field of view (FOV)/field of perception (FOP) is measured in degrees (diagonal/vertical/horizontal).

Sensor	Resolution	Frame Rate	FOV ^a^/FOP ^b^	Range
RealSense ZR300 ^d^				
Depth ^c^	628 × 468, 320 × 240	30, 60	80/60/60	0.55 m, 2.8 m
Infrared (two cameras)	640 × 480, 332 × 252	30, 60	70/46/59	-
Colour (RGB)	1920 × 1080, 640 × 480	30, 60	75/41.5/68	-
Fisheye (monochrome)	640 × 480	60	166.5/100/133	-
RealSense D435 ^e^				
Depth ^c^	1280 × 720, 848 × 480	30, 90	99/63/90	0.28 m, 3 m
Infrared (two cameras)	1280 × 720, 848 × 480	30, 90	95/58/87	-
Colour (RGB)	1920 × 1080, 960 × 540	30, 60	77/42/69	-

^a^ Field of view. Angular extent covered by a camera. ^b^ Field of projection. Angular extent covered by a projector (e.g., laser). ^c^ Infrared laser. ^d^ Official designation: Intel©RealSense™ZR300. ^e^ Official designation: Intel©RealSense™D435.

**Table 7:** Leave-one-recording-out cross-validation results (i.e., concordance correlation coefficient (CCC) and Pearson correlation coefficient (r)) using linear regression (LR) and random forest (RF). We also report results of self-reported emotion by Trung et al. [51] for comparison.

Regression	Method	CCC	*r*
Valence	LR	0.113	0.338
	RF	0.237	0.256
	Trung et al. [51]	–	0.18
Arousal	LR	-0.046	0.123
	RF	0.0230	0.0256
	Trung et al. [51]	–	0.25

**Table 8:** Segmentation error rate and diarisation error rate of the meetings’ corpus.

Method	$P_{k}$	WD	DER
vad 0	32.34%	56.45%	99.58
$vad_{offset}$	32.09%	55.48%	-
auditok	35.91%	61.86%	95.59
lium	44.49%	51.43%	100.35
auditok + vad	32.44%	74.19%	99.89
lium + vad	38.44%	66.30%	108.45

## Data Availability

The data will be made available on request to SL.
